# Indicators to assess the quality of programs to prevent occupational risk for tuberculosis: are they feasible?

**DOI:** 10.1590/1518-8345.0591.2695

**Published:** 2016-06-07

**Authors:** Talita Raquel dos Santos, Maria Clara Padoveze, Lúcia Yasuko Izumi Nichiata, Renata Ferreira Takahashi, Suely Itsuko Ciosak, Anna Luiza de Fátima Pinho Lins Gryschek

**Affiliations:** 1 Master's Student, Escola de Enfermagem, Universidade de São Paulo, São Paulo, SP, Brazil. RN, Hospital Universitário, Universidade de São Paulo, São Paulo, SP, Brazil.; 2 PhD, Professor, Escola de Enfermagem, Universidade de São Paulo, São Paulo, SP, Brazil.; 3 Associate Professor, Escola de Enfermagem, Universidade de São Paulo, São Paulo, SP, Brazil.

**Keywords:** Tuberculosis, Quality Indicators, Health Care, Occupational Risk

## Abstract

**Objective:**

to analyze the feasibility of quality indicators for evaluation of hospital programs for preventing occupational tuberculosis.

**Method:**

a descriptive cross-sectional study. We tested indicators for evaluating occupational tuberculosis prevention programs in six hospitals. The criterion to define feasibility was the time spent to calculate the indicators.

**Results:**

time spent to evaluate the indicators ranged from 2h 52min to 15h11min 24sec. The indicator for structure evaluation required less time; the longest time was spent on process indicators, including the observation of healthcare workers' practices in relation to the use of N95 masks. There was an hindrance to test one of the indicators for tuberculosis outcomes in five situations, due to the lack of use of tuberculin skin test in these facilities. The time requires to calculate indicators in regarding to the outcomes for occupational tuberculosis largely depends upon the level of organizational administrative structure for gathering data.

**Conclusions:**

indicators to evaluate the structure for occupational tuberculosis prevention are highly feasible. Nevertheless, the feasibility of indicators for process and outcome is limited due to relevant variations in administrative issues at healthcare facilities.

## Introduction

Tuberculosis continues to be a threat worldwide. Consequently, many healthcare workers (HCW) are at risk of being infected and acquiring this disease^1^. Good prevention programs should be established to avoid this undesirable outcome in healthcare facilities. To evaluate such programs, quality indicators can be used to identify the level of compliance for recommended practices.

Quality indicator technology has been increasingly used for evaluating health care practices. They are quantitative measures of features or attributes of a given process or system^2^, which may indicate the heath care quality provided, as well as specific needs for improvement^3^. Three classical categories have been used for their classification: structure, process and outcome^2^ ^,^ ^4^.The advantage of one over the other lies in the characteristics of the phenomenon to be measured. 

*Structure* indicators refer to the features required, such as human resources, equipment, information systems, etc. *Process* indicators measure the dynamics of a given process, or how this particular process was performed. *Outcome* indicators measure the frequency in which event occurs, and assess final goals, such as mortality, morbidity or patient satisfaction^2^ ^,^ ^5^. Ideal indicators include features such as acceptability, objectivity, effectiveness, reliability, feasibility and availability, communication, interpretability, reproducibility, context, sensitivity to change, efficiency, and comparability^6^. 

In 2006, a group of researchers in Brazil constructed and validated a set of indicators designed to evaluate the quality of programs for healthcare-associated infection (HAI) prevention, including occupational tuberculosis. They can also be used to gauge the extent to which the control of HAI differs between different institutions^5^ ^,^ ^7^. Although the content was validated by professional experts, these indicators have not yet been fully tested. 

Due to great difficulty in finding patterns for feasibility assessment in the literature, the best criteria for defining feasibility was previously discussed in a focus group with specialists^6^ ^,^ ^8^. The criterion "time" was chosen as a way of classifying these indicators as feasible. Providing the extent of time spent in measuring the indicator is as short as possible, the indicator is considered feasible. The shorter the time, the lower the human resources expense, and the more likely the indicator is to be widely use.

In the present study we aimed to analyze the feasibility of these quality indicators for the evaluation of programs for preventing occupational tuberculosis. 

## Methods

This descriptive, cross-sectional study tested the feasibility of quality indicators aimed at evaluating elements of structure, process and outcome of occupational tuberculosis prevention programs in different healthcare facilities. Time required for the calculationof the indicators was assessed as a measure of feasibility.

**Figure 1:**
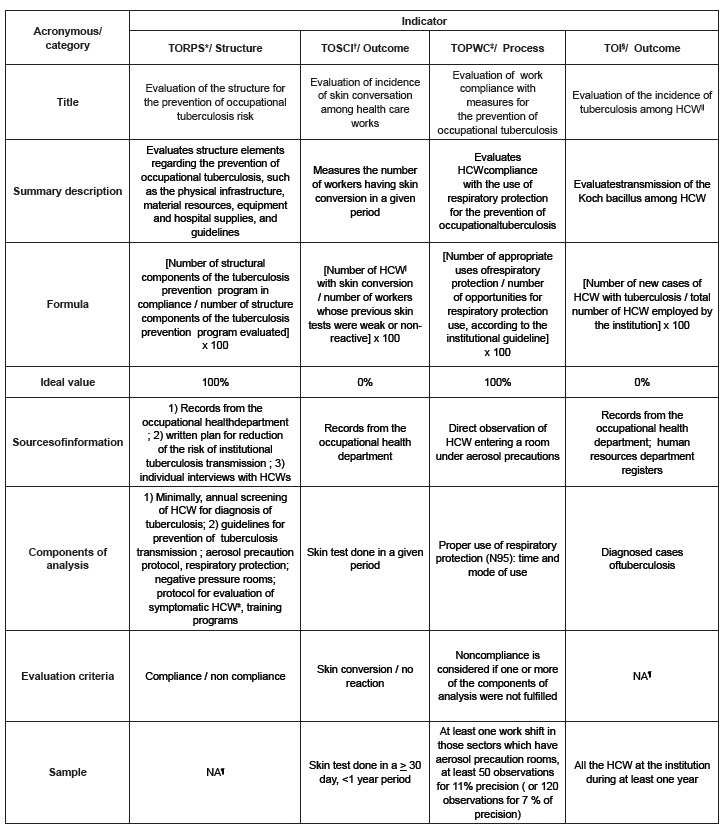
Indicators for evaluating prevention and control programs for biological occupational risk of tuberculosis, according to Takahashi^7^. São Paulo, SP, Brazil, 2011

The quality indicators are described in *Figure 1*, with a brief description, formula, ideal values, sources of information, components of analysis, evaluation criteria and sample.

* Tuberculosis Occupational Risk Prevention Program Structure.† Tuberculosis Occupational Skin Conversion Incidence.‡Tuberculosis Occupational Prevention Workers Compliance.§ Tuberculosis Occupational Incidence. ||Health Care Workers ¶Not applicable.

Indicators were applied in six different institutions in the city of São Paulo, Brazil, which met the following requirements: a) acute care hospital, b) public or private setting, c) caring for patients with suspected or confirmed pulmonary or laryngeal tuberculosis in the bacillary phase, and d) having a formal Healthcare-associated Infection Control Committee (HICC).

Selected variables were used to characterize the participant institutions and to identify the components that may contribute to the variations in the time required to calculate the indicators. These variables included the number of active beds; the average prevalence of daily (or monthly) inpatients with pulmonary orlaryngeal tuberculosis bacillus and aerosol precautions indicated, number of employees in the institution, and nature of the institution (public / private / philanthropic).

Data were collected using a standardized form, and the time was measured using a chronometer. Secondary variables were collected in order to identify elements justifying the time spent to collect each indicator. Periods of interruption and time intervals were deducted from the overall time span of the activity. Also, the time spent on healthcare facility characterization, as well as the time required to access the hospital facilities (reception, elevators, etc.) was not included in data collection. Indicators were tested by the same researcher in all healthcare facilities (T.R.S.).

The variables of analysis were the time spent on: a) data collection, and b) data consolidation and analysis. We compared the time spent on testing of each indicator in the different institutions.

Data were collected from December 2010 to July 2012. This period was required to complete data collection in all six institutions, due to the small number of inpatients having pulmonary or laryngeal tuberculosis who were placed in aerosol precautions in some of the hospitals.The data analysis was descriptive.

## Results

Among the six institutions in which the quality indicators were tested, four were general hospitals, one was a hospital specializing in infectious disease, and one was a general hospital, although it served as a reference site for tuberculosis treatment (Table 1). Among 2,655 beds in six institutions, 55.54% (1,480) were public. Altogether, these institutions had approximately 24,271 health workers, 45.91% (11,145) in the public sector. Among the 690 hospitalized patients with diagnosed or suspected pulmonary or laryngeal tuberculosis, 94.63% (653) were admittedtopublic institutions.

**Table 1:** Characteristics of the institutions surveyed and time spent for collection and consolidation of quality indicators for occupational tuberculosis prevention programs. São Paulo, SP, Brazil, 2011-2012

Institution						
Characteristics	A	B	C	D	E	F
Number of active beds Number of patients with laryngeal or pulmonary tuberculosis bacillus, hospitalized in the last year, indicating isolation.	220	983	341	220	614	277
	489	96	11	11	15	68
Number of health workers in the institution	1737	6000	4126	1300	9700	1408
Natureoffunding	Public	Public	Private	Private	Private	Public
Time required for data collection and consolidation of indicator (h/min/sec)						
TORPS^*^	00:24:39	00:18:38	00:17:56	00:12:19	00:23:54	00:18:25
TOI^†^ TOSCI^‡^	01:02:03	00:19:40	-	00:04:15	00:02:52	00:03:27
	-	00:06:58	-	-	-	-
TOPWC^§^	04:44:28	04:56:15	14:23:13	15:11:24	08:37:49	11:44:58
Data consolidation	01:17:57	01:13:25	01:00:07	01:04:30	00:58:39	01:01:08
Total time	07:29:08	06:55:33	15:51:16	16:32:28	10:03:14	13:17:58

* Tuberculosis Occupational Risk Prevention Program Structure.**^†^** Tuberculosis Occupational Incidence.**^‡^** Tuberculosis Occupational Skin Conversion Incidence.^§^Tuberculosis Occupational Prevention Workers Compliance.

 All evaluated facilities had the same recommendation for the use of a N95 particulate respirator: to put it on in the anteroom or in the hallway before entering the room of a patient with known or suspected pulmonary or laryngeal tuberculosis bacillus.

The TORPS indicator resulted in minimal effort and time spent on its application in all institutions (Table 1) Regarding the TOSCI indicator, the information necessary for its calculation was not found in five of the six health institutions. Several arguments were used to report the absence of the use of Tuberculosis Skin Test (TST): lack of trained personnel to perform the test, the porosity of collection, frequent lack of HCW follow-up for appropriate characterization of the reaction; difficulty in identifying the exact time period of the HCW's exposure to the mycobacterium. 

The TOI indicator was collected in five of the six institutions; there was only one healthcare facility in which data were not organized in such a way that it could be collected. The time used to collect this indicator was not toolong, but depended on the level of data organization. 

The TOPWC indicator required greater time for calculation(Table 1)*.* To note, public hospitals were more likely to require less time to collect data than the private sector. In public hospitals there are usually more patients admitted with tuberculosis, therefore it was possible to observe two or more patients simultaneously, thus reaching 51 observations more rapidly.

## Discussion

Many quality indicators have been proposed in the literature, however few have been evaluated regarding their feasibility for application, which creates a gap between theory and practice. Nevertheless, the recommendation for their use is quite frequent. To our knowledge, the present study is the first to evaluate the feasibility of quality indicators, using as the criterion the time spent on administering / calculatingthem.

Information on quality of care depends upon data availability. Therefore, quality is difficult to measure without correct and consistent information, which is often unavailable^8^. A previous study evaluated the feasibility of quality indicators related to radical prostatectomy and concluded that indicators not obtaining more than 25.9% of the necessary information were considered unenforceable^9^. It has also been previously shown that quality indicators for antibiotic treatment of complicated urinary tract infections were considered feasible if the data necessary to score the indicator can be abstracted from the available data for >70% of cases^10^. Indicators should require ease of obtaining data or ease of availability of the data as a condition of feasibility, resulting in minimal effort and additional cost^6^ ^,^ ^11^. Because time spent on data gathering and analysis reflects both on efforts and cost, less time means higherf easibility.

Although time spent on the application of quality indicators of an occupational tuberculosis prevention program may vary in different healthcare facilities, some common features were noted from this study. For instance, the indicator that evaluated the structure of the program (TORPS) proved to be highly feasible. This indicator has characteristics suggestive of being used for external audits and evaluations. On the other hand, the process indicator (TOPWC) requires greater dedication of professional time for its application. This indicator should be used preferentially by healthcare facilities that have a higher number of in-patients requiring special precautions for tuberculosis, aiming to evaluate compliance with the use of the N95 mask by HCWs. As a suggestion, TOPWC could be applied biannually, or after major intervention and training programs.

It is a matter for discussion as to why, despite recommendations, some healthcare facilities in Brazil are not using the TST routinely, as we demonstrated in our sample. As an outcome to be measured, it was shown that the indicator for skin conversion (TOSCI) was not feasible due to this lack of compliance. The Centers for Disease Control recommends the use of the TST whenever there is the possibility of high exposure to tuberculosis^12^. HCWs should be periodically screened for latent tuberculosis infection using TST. As pointed out, concerning the healthcare facilities, many operational issues can interfere in the process. Among these issues, are the high turnover of HCWs, the limitations of the TST interpretation, and a potential booster effect of the BCG vaccine^13^ ^-^ ^15^.In order to overcome the booster effect, a two-step TST has been suggested in the literature^15^ ^-^ ^17^.The TST has a high sensitivity, but lacks specificity in a vaccinated population, such as the HCWs in Brazil. Due to this feature, countries such as France and Japan are now recommending, with some restrictions, the gama-interferon release assays as a substitute for TST^18^ ^-^ ^19^. To note, in our sample, none of the healthcare facilities that were not using TST provided any other screening measure as a substitute. 

The main outcome indicator (TOI), which measures the incidence of cases of tuberculosis among HCW, is quite simple to obtain, provided the Occupational Medicine Service has a structured form to record such cases. Usually cases of occupational tuberculosis are not as frequent as to warrant a great deal of effort in recording them. Besides this, the number of exposed HCWs is, in general, quite steady and does not require a sophisticated system to collect the information. Despite this, many healthcare facilities are not aware of monitoring the annual incidence of occupational cases of tuberculosis. 

The World Health Organization (WHO) shows that tuberculosis mortality in Brazil in 2013 was 3.2/100.000 and the prevalence was 57/100.000^20^. Some authors have published similar results. A Peruvian study found a tuberculin test conversion incidence in medical students of approximately 3%^21^. A Brazilian study conducted in Belo Horizonte, MG, Brazil, where the tuberculosis incidence rate is 23/100.000, had the cooperation of 251 HCWs. The TST conversion was 5.1%, with the risk of infection of 1.4^22^. A study aimed to identify the TST conversion rate of HCWs with previously negative TST results who had been working for less than 1 year in a hospital in Botswana, where tuberculosis is highly endemic. This population had a conversion rate of 4.2% for the entire group studied, or 6.87 per 1000 person-weeks^23^.

A Chinese study showed that the health care workers' annual tuberculosis notification rates were lower than the general population. Healthcare workers with tuberculosis were a mean of 35.5 years old, with females out numbering males (58.0%>42.0%). The proportion of pulmonary tuberculosis was significantly higher among the women compared with men (88.5%>83.4%, p = 0.031). This study suggested that the priority for tuberculosis prevention in healthcare institutions should be given to the young female HCWs^1^.

 An Argentinean study that included 15,276 HCWs from 15 centers found a mean incidence rate of tuberculosis in 111.3/100,000 HCWs^24^; A Brazilian study demonstrated incidence rates in the general population of approximately 62/100,000, a prevalence of tuberculosis infection in HCW of 63.1% and an annual rate of tuberculin conversion of 10.7%^25^. In such an epidemiologic context, monitoring the incidence of occupational tuberculosis and the TST conversion can aid institutions in planning and evaluating strategies for occupational tuberculosis prevention, as demonstrated by other authors^13^ ^,^ ^15^.

With 1.5 million deaths in 2013 and 5.7 million new cases of tuberculosis disease, the WHO goal is to dramatically reduce the global burden of tuberculosis by 2015^20^. For this control, it will be necessary to include the successful development and application of new drugs, diagnostics, vaccines, and prevention tools as well as a clearer understanding of the impact of social and economic determinants of this disease in the health sector. The quality indicators of programs for prevention of occupational tuberculosis evaluated in the present study were shown to be feasible. Since HCWs have 2- 50 times the chance of acquiring the disease than people in the general population, these indicators can help institutions prevent occupational tuberculosis. Therefore, we recommend their application at least once a year in healthcare facilities that frequently deal with patients affected by tuberculosis.

The results are limited by the small amount of participant institutions, which only enables a suggestion of possible relationships between indicators and the institutional profile. Further studies should include multiple institutions to enable the investigation of relationships between the nature of the institution and the feasibility of applying the quality indicators. , There were not many objective criteria found in the literature that allowed for the evaluation of the applicability of indicators, so it was decided to use time as a marker. However, we understand that this is a specific perspective that limits the study.

This study brings new insight to the applicability of previously validated quality indicators, revealing that even a validated indicator may not have all the properties of applicability; this approach needs to be considered to suggest recommendations for their use.

Moreover, strengths in the structure assessment, and weaknesses in the process and outcomes assessments, have been identified. Areas to be improved include maintaining periodic screening for latent tuberculosis using TST, monitoring the annual incidence of occupational cases of tuberculosis, and evaluating compliance with occupational prevention.

## Conclusion

The indicators to evaluate the structure for occupational tuberculosis prevention are highly feasible. The feasibility of applying indicators for process and outcome is limited, due to relevant differences in administrative issues at healthcare facilities, such as the system for data archiving and management.
